# Time fractional Yang–Abdel–Cattani derivative in generalized MHD Casson fluid flow with heat source and chemical reaction

**DOI:** 10.1038/s41598-023-43630-9

**Published:** 2023-10-01

**Authors:** Sehra, Haleema Sadia, Sami Ul Haq, Ilyas Khan

**Affiliations:** 1 Shaheed Benazir, Bhutto Women University Peshawar, Peshawar, Khyber Pakhtunkhwa 25000 Pakistan; 2 Department of Mathematics, Islamia College Peshawar, Peshawar, Khyber Pakhtunkhwa 25000 Pakistan; 3 Department of Mathematics, College of Science Al-Zulfi, Majmaah University, Al-Majmaah, 11952 Saudi Arabia

**Keywords:** Mathematics and computing, Physics

## Abstract

This present research article investigates the exact analytical solution for the mathematical model of the generalized Casson fluid flow by using the new fractional operator with Rabotnov exponential kernel i.e. Yang–Abdel–Cattani operator. The impacts of heat source, magnetic hydrodynamics and chemical reactions on the flow of fractional Casson fluid through a vertical flat plate are studied in this article. For the sake of a better interpretation of the rheological behavior of Casson fluid we have used the new operator of fractional order with exponential kernel of Rabotnov known as Yang–Abdel–Cattani operator of fractional derivative. By making use of the technique of Laplace transform we have find the exact analytical solution of the problem in the Mittag–Leffler’s form, for all the three governing equations i.e. Velocity, energy and concentration equation. It has been noticed from the literature that it is challenging to obtain analytical results from fractional fluid model derived by the various fractional operators. This article helps to address this issue by providing analytical solutions for fractionalized fluid models. To analyze the physical importance of different fluid parameters such as Schmidt number, Prandtl number, MHD and alpha on the heat, mass and momentum class are presented through graphs. The concentration of the fluid decreases with Schmidth number and temperature of the fluid decreases with the increasing Prandtl number. The velocity of the fluid decreases with increasing MHD effects and increases with increasing Alpha. The Yang–Abdel–Cattani operator of fractional order can describe the memory effects more suitably than the other fractional operators.

## Introduction

The process of transfer of mass and heat has many important applications in the industrial field. A large number of scientists and researchers worked on this area. The theory of Non-Newtonian fluid has a great impact on the modern technologies and different industrial field due to the failure of the Newtonian fluid theory in the expression of various flow characteristics. The simulation and modeling of the Non-Newtonian fluid flow play a significant role in our daily life and facilitated the Human life in different ways. Many researchers presented various models for the Non-Newtonian fluid flows i.e. Maxwell model, Oldroyd-B, Walters-B, Casson, Brinkman type, Jeffery, Bingham plastic, power law, visco-plastic fluid and second grade model to study the physical and computational characteristics of the fluid^1–3^.

Several fluid models presented previously in the literature have many drawbacks and limitations such as, second grade model of fluid flow cannot explains the viscosity but this model efficiently explained the elasticity in fluid, the Power law model fails to explain the elasticity effects but explained the viscous properties of the fluid properly, this attract many mathematicians and researchers to study such complex fluids. The systematic study of this type of fluid flow is very important in the theoretical analysis as well as in practical implementation of modern machinery. Among these fluid models, the most common Non-Newtonian model which is also known as shear-thinning liquids i.e. Casson fluid model attracted special attention because of its applications and significant role in various fields such as chemical and mechanical applications, metallurgy and its application in industries of fluid processing.

An important property of Casson fluid Model is that it describes two different matter states. It adopts the behavior of a solid having elasticity in the case when the applied tangential force in comparison to the yield stress is less, So no flow occurs in this case, and the flow takes place in the case when the yield stress in comparison to the shear stress is less. Some daily life examples of Casson fluid are synthetic lubricants, artificial fibers, concentrated fruit juices, tomato sauce, honey, jelly, soup, paints and coal, china clay and pharmaceutical chemicals. The Blood in the living bodies is considered as Casson fluid because it contains various materials such as fibrinogen, globulin and protein in the red blood cells and plasma with aqueous base in^4,5^. Initially Casson in 1959 presented the model of the Casson fluid for the estimation of the pattern of flow for the pigment-oil suspensions^6^. Several engineers, researchers, scientists and mathematicians explored the properties of the Casson model of fluid in relation to the fluid mechanics dependent upon different situations^7^.

In^8^ Khalid et al. investigated the unsteady and natural convection during the Casson fluid flow with magnetic hydrodynamic impacts in a porous medium. In^9^ Bhatta charyya et al. studied the Casson fluid flowing via a stretching and shrinking plane with the impacts of magnetic hydrodynamics. In^10^ Oka and Syoten analyzed the Casson fluid flowing in tubes for the first time. In^11^ Mernone et al. analyzed the Casson fluid peristaltical flows in a two-dimensional channel. Arthur et al.^12^ investigated the deformation occurring during the flow of Casson fluid in a medium having porosity with the effects of chemical reaction and induced magnetic field. In12] Mukhopadhyay investigated the impacts of heat radiation and suction of heat on temperature over a stretching surface during the Casson fluid flow. Mustafa et al.^13^ explained the heat transport phenomenon in a Casson fluid flowing through a flat plate that is in motion by applying homotopy technique of investigation for the unsteady flow in the boundary layer. In^14^ the author used an organized approach to analyze the influence of radiative thermal flux on mass and heat transference in a medium having porosity.

The main objective behind, finding the exact numerical solutions is its important applications in different field in our daily life^15,16^. To obtain exact numerical solution, numerous techniques are implemented by mathematicians and researchers. For instance, residual power series method^17^, simple equation modification method^18^, reproducing the kernel Hilbert space method^19,20^, Riccati-Bernoulli Sub-ODE technique for sub-ordinary differential equation (RBSODET)^21^, multi steps approach^22,23^, unified method^24^ and several others^25–27^. As a result of advancements in the subject, Scientists have proposed a few new methods to describe and establish the solution to real world problems using the concept of fractional (non-integer order) calculus. To describe and design the model for different flow patterns in several fields for example fractal rheological models, models for electric circuit and populations fractal growth models, a lot of operators of fractional order have non-singular kernel but some of these operators have singular kernel. These Fractional operators are very important tool for the analysis of rheological properties of different physical models. In the literature, numerous researchers work hard to analyze fractional fluid models and derive a variety of compelling results which are useful for scientists and engineers to compare their experimental outcomes obtained from the governing PDE’s with the analytical results gathered making use of various mathematical tools and techniques of fractional models of the non-Newtonian fluid^28–30^. Fractional integrals and fractional derivatives operators, both invented by Marchaud Caputo and Riemann–Liouville are based on singular kernels, have several limitations. For example, the modeling process for these fractional models was very challenging.

To minimize the difficulties that occurs in singular fractional models, some models are presented with non-singular exponential kernels such that fractional operators of Prabhakar, Yang Abdel Cattani, Caputo-Fabrizio, Atangana-Baleanu and some others described in^31–33^. Some of the kernels of these non-singularized fractional operators are Exponential kernels, Mittag–Leffler functions and Rabotnov exponential function. In^34^ the authors analyzed the Casson fluid flow without the consideration of mass transfer by using Caputo fractional model and obtained the analytical solution of the problem by applying integral Laplace transform, because of its efficient application for the conditions of non-uniform boundary. In^35^ the authors analyzed the time fractional model of the Casson fluid based on the generalized Fourier’s and Fick’s Laws by using new fractional operator with Rabotnov exponential kernel i.e. Yang–Abdel–Cattani operator of fractional order.

The time fractional analysis by the Yang–Abdel–Cattani has many industrial applications for example in the field of medial it is used to design a mathematical model for the growth of the tumor cells with chemotherapeutic cells^36^. The time fractional approach is used to analyze of stuxnet virus fractional growth in the industrial control system^37^. The time fractional approach is used to model the electrochemical double layer capacitors mathematically^38^. It has been noticed from the literature that it is challenging to obtain analytical results from fractional fluid model derived by the various fractional operators. This article helps to address this issue by providing analytical solutions for fractionalized fluid models.

The authors studied the general form of the fractional reduced differential transform method FRDTM to the (N + 1)-dimensional cases and applied this method to handle the time fractional couple Whitham–Broer–Kaup’s type system in^39^. The authors analyzed the Hausdorff vector calculus based on the Chen Hausdorff calculus for the first time and obtained Stokes-like, Gauss–Ostrogradsky-like and Green-like theorems, and Green-like identities in the framework of the Hausdorff vector calculus in^40^. The authors considered some fractional integral formulas in terms of the Riemann–Liouville, Erdélyi–Kober type, and Weyl fractional integral operators and present the general fractional kinetic model involving the hyper geometric super hyperbolic sine function via the Gauss hyper geometric series in^41^. The authors addressed a novel anomalous relaxation model with the new general fractional derivative of the Sonine kernel^42^. The authors defined a weighted Caputo-type differential operator which was used to character relaxation and diffusion models in two different types. Then, one of the weighted Caputo-type integral operators by solving the related linear differential equation was also defined in^43^.

In the published literature the fractional model of the Casson fluid with symmetric conditions for heat, concentration and momentum with the impacts of heat source, magneto hydrodynamics and chemical reaction and are until now neither studied nor published. To address this gap we solve a model of fractional Casson fluid with suitable conditions on concentration, temperature and velocity distributions. We use the new fractional operator with the Rabotnov kernel known as Yang–Abdel–Cattani operator to fractionalize velocity, temperature and concentration equations. As we are interested in the rheology of Casson fluid, to study it betterly we use the new fractional operator with exponential kernel of Rabotnov functions i.e. Yang–Abdel–Cattani fractional derivative as it can describe the generalized memory effects very well. To obtain the exact analytical solution of the problem in the form of Mittag–Leffler functions the method of Laplace transform is used. To visualize the influence of physical parameters on the momentum, temperature and mass of the fluid such as Prandtl number Pr, thermal Grashof number Gr, MHD, fractional order of YAC and Schmidt number, the effects are presented graphically.

### Mathematical model

**Figure 1:**
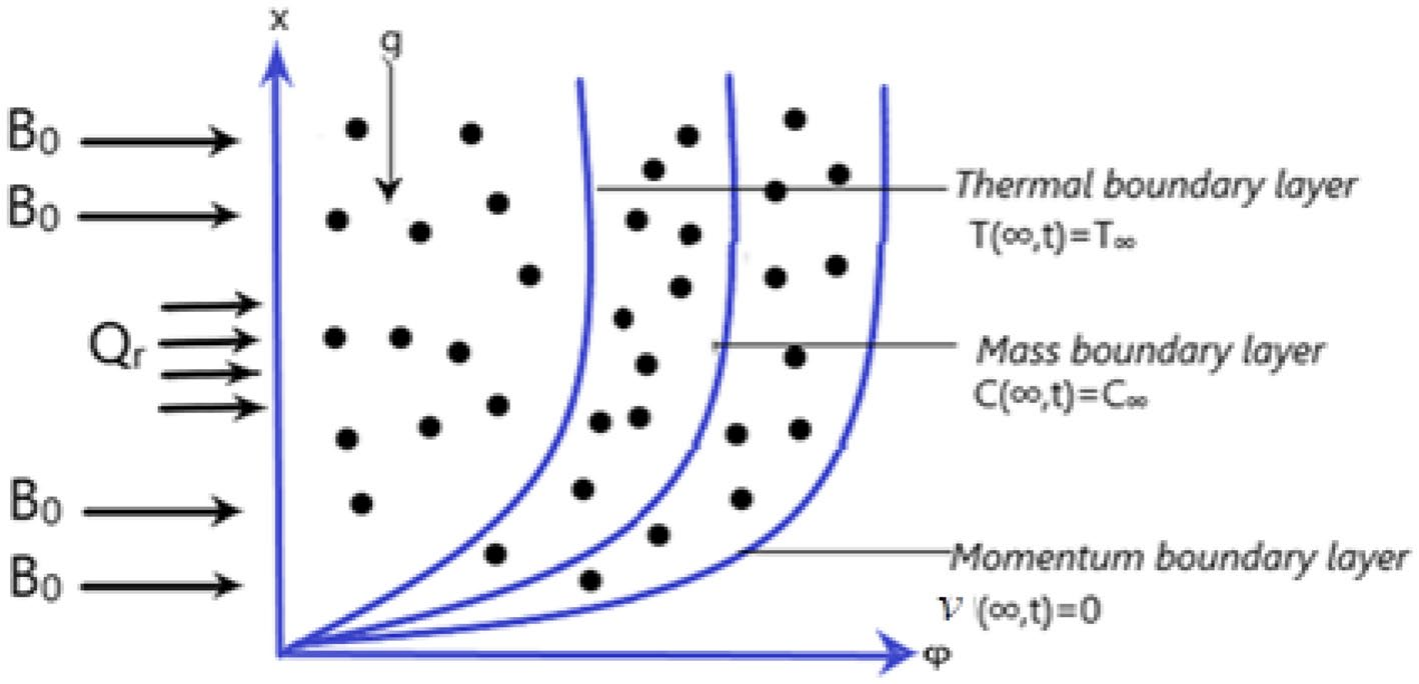
flow Geometry of the model.

Take into consideration a Casson fluid flowing through an infinite plate under the influence of magneto hydrodynamics. The influence of source of heat at boundary and chemical reaction are also considered. In start at some time t = 0 the plate through which the fluid is flowing and the flowing fluid both are stationary with the constant temperature ${\mathrm{T}}_{\infty }$ and constant concentration ${\mathrm{C}}_{\infty }$. At some later time t = ${0}^{+}$ the ramping wall conditions are considered for the temperature and velocity such that φ = 0 the concentration is C(0,t) = ${\mathrm{C}}_{\mathrm{w}}$, the wall temperature is ${\mathrm{T}}_{\mathrm{w}}$ and with the characteristic velocity ${\mathrm{u}}_{0}$ the fluid’s velocity along x-axis is given as $v$(φ, t). The Ramped wall condition on the Casson fluid flow has several important applications in different field such as modern industrial field and medical sciences. The fluid’s velocity obeys the continuity equation under the effects of assumed factors. By taking into consideration the given supposition, we obtain the following governing equations for the energy, mass and velocity of the fluid by using the Boussinesq’s approximation^35^. Figure 1 shows the geometry of the flow dynamics.

Momentum equation;

1 $$\rho \frac{\partial v(\varphi ,t)}{{\partial t}} = \mu \left( {1 + \frac{1}{\gamma }} \right)\frac{{\partial^{2} v(\varphi ,t)}}{{\partial \varphi^{2} }} - \sigma B_{0}^{2} v(\varphi ,t) + \rho g\beta_{T} \left( {T(\varphi ,t) - T_{\infty } } \right) + \rho g\beta_{C} \left( {C(\varphi ,t) - C_{\infty } } \right),$$

Temperature equation;

2 $$\rho C_{p} \frac{\partial T(\varphi ,t)}{{\partial t}} = - \frac{\partial q(\varphi ,t)}{{\partial \varphi }} - Q\left( {T(\varphi ,t) - T_{\infty } } \right){,}$$

With generalized Fourier’s law

$$q(\varphi ,t) = - K\frac{\partial T(\varphi ,t)}{{\partial \varphi }}{,}$$

Diffusion equation;

3 $$\frac{\partial C(\varphi ,t)}{{\partial t}} = - \frac{\partial \chi (\varphi ,t)}{{\partial \varphi }} - k\left( {C(\varphi ,t) - C_{\infty } } \right),$$

With generalized Fick’s law;

$$\chi (\varphi ,t) = - D_{m} \frac{\partial C(\varphi ,t)}{{\partial \varphi }},$$

With the considered IC’s and BC’s are given as;

4 $$\begin{aligned} & v(\varphi ,0) = 0,\;\;T(\varphi ,0) = T_{\infty } ,\;\;C(\varphi ,0) = C_{\infty } ,\;\;\varphi \ge 0, \\ & v(0,t) = u_{0} ,\;\;T(0,t) = T_{w} ,\;\;C(0,t) = C_{w} ,\;\;t > 0, \\ & v(\varphi ,t) \to 0,\;\;T(\varphi ,t) \to T_{\infty } ,\;\;C(\varphi ,t) \to C_{\infty } \;\;as\;\;\varphi \to \infty . \, \\ \end{aligned}$$

The following set of dimensionless variables is introduce to non-dimensionalize the system equation;

5 $$\begin{aligned} & t^{ * } = \frac{{u_{0}^{2} t}}{\nu },\;\;\varphi^{ * } = \frac{{u_{0} \varphi }}{\nu },\;\;{\text{v}}^{ * } = \frac{v}{{u_{0} }},\;\;\nu = \frac{\mu }{\rho },\;\;T^{ * } = \frac{{T - T_{\infty } }}{{T_{w} - T_{\infty } }},\;\;C^{ * } = \frac{{C - C_{\infty } }}{{C_{w} - C_{\infty } }}, \\ & Gr = \frac{{g\beta_{T} \nu (T_{w} - T_{\infty } )}}{{u_{0}^{3} }},\;\;Gm = \frac{{g\beta_{c} \nu (C_{w} - C_{\infty } )}}{{u_{0}^{3} }},\;\;\Pr = \frac{{\mu C_{p} }}{k},\;\;\frac{1}{Sc} = \frac{D}{\nu }, \, \\ & \left( {1 + \frac{1}{\gamma }} \right) = \frac{1}{\lambda },\;\;{\text{M = }}\frac{{\delta B_{0}^{2} }}{{u_{0}^{2} \rho }},\;\;\eta_{1} { = }\frac{{\nu^{2} Q}}{{u_{0}^{2} k}},\;\;q^{*} = \frac{q}{{q_{0} }},\;\;\eta_{2} = \frac{k\nu }{{u_{0}^{2} }}. \\ \end{aligned}$$

To achieve the non-dimensional form of the equation we use Eq. (5) in (1–3) and then ignored the esterisk sign from the dimensionless variables we got the following model.

Momentum equation;

6 $$\frac{\partial v(\varphi ,t)}{{\partial t}} = \frac{1}{\lambda }\frac{{\partial^{2} v(\varphi ,t)}}{{\partial \varphi^{2} }} - Mv(\varphi ,t) + GrT(\varphi ,t) + GmC(\varphi ,t),$$

Temperature equation;

7 $$\frac{\partial T(\varphi ,t)}{{\partial t}} = - \frac{1}{\Pr }\frac{{\partial^{2} T(\varphi ,t)}}{{\partial \varphi^{2} }} - \eta_{1} T(\varphi ,t),$$

With dimensionless generalized Fourier’s law;

$$q(\varphi ,t) = - \frac{\partial T(\varphi ,t)}{{\partial \varphi }}{,}$$

Concentration equation;

8 $$\frac{\partial C(\varphi ,t)}{{\partial t}} = - \frac{1}{Sc}\frac{{\partial^{2} C(\varphi ,t)}}{{\partial \varphi^{2} }} - \eta_{2} C(\varphi ,t),$$

With dimensionless generalized Fick’s law;

$$\chi (\varphi ,t) = - \frac{\partial C(\varphi ,t)}{{\partial \varphi }}.$$

After the dimensionless analysis the IC’s and BC’s are given as;

9 $$\begin{aligned} & v(\varphi ,0) = 0,\;\;T(\varphi ,0) = 0,\;\;C(\varphi ,0) = 0,\;\;\varphi \ge 0, \\ & v(0,t) = 1,\;\;T(0,t) = 1,\;\;C(0,t) = 1,\;\;t > 0, \\ & v(\varphi ,t) \to 0,\;\;T(\varphi ,t) \to 0,\;\;C(\varphi ,t) \to 0\;\;as\;\;\varphi \to \infty . \\ \end{aligned}$$

### Some basic results

The Yang–Abdel–Cattani derivative of fractional order 0 < α < 1 is described as:

10 $$^{YAC} D_{t}^{\alpha } f(t) = \int\limits_{0}^{t} {\psi_{\alpha } \left( { - \wp (t - \tau )^{\alpha } } \right)} f^{\prime}(\tau )d\tau \;\;{\text{for}}\;\;{\text{t}} > {0}\;\;{\text{and}}\;\;{0} < \alpha < 1.$$

where;

11 $$\psi (\wp z^{\alpha } ) = \sum\limits_{n = 0}^{\infty } {\frac{{\wp^{n} z^{(n + 1)(\alpha + 1) - 1} }}{\Gamma (n + 1)(\alpha + 1)}\;\;{\text{for}}\;\;{\text{z}} \in {\mathbb{C}}} {,}$$

where $\psi_{\alpha }$ describes the exponential kernel of Rabotnov of fractional order α such that $\mathrm{\alpha }\in (\mathrm{0,1})$.

The Laplace integral transform of the Yang–Abdel–Cattani operator of fractional order derivative is given as;

12 $$L\left\{ {^{YAC} D_{t}^{\alpha } f(t)} \right\} = \frac{1}{{s^{\alpha + 1} }}\frac{{sL\{ f(t)\} - f(0)}}{{1 + \wp s^{ - (\alpha + 1)} }}{.}$$

where s is the parameter of the Laplace integral transform and $\alpha$ is the fractional parameter of Yang–Abdel–Cattani operator of derivative.

The Yang–Abdel–Cattani operator of fractional order, a unique mathematical model that generalize the effects of heat memory, is introduced in this article. Based on the Yang–Abdel–Cattani operator of fractional order derivative, the following is the Casson fluid’s time-fractional model for mass, energy, and momentum:

13 $$^{YAC} D_{t}^{\alpha } v(\varphi ,t) = \frac{1}{\lambda }\frac{{\partial^{2} v(\varphi ,t)}}{{\partial \varphi^{2} }} - Mv(\varphi ,t) + GrT(\varphi ,t) + GmC(\varphi ,t),$$

14 $$^{YAC} D_{t}^{\alpha } T(\varphi ,t) = \frac{1}{\Pr }\frac{{\partial^{2} T(\varphi ,t)}}{{\partial \varphi^{2} }} - Q_{0} T(\varphi ,t),$$

15 $$^{YAC} D_{t}^{\alpha } C(\varphi ,t) = \frac{1}{Sc}\frac{{\partial^{2} C(\varphi ,t)}}{{\partial \varphi^{2} }} - \eta_{2} C(\varphi ,t).$$

### Solution of the problem

To acquire an exact analytical solution to the problem of the time fractional Casson fluid model, we will apply the Laplace integral transform to (13–15) and use the result presented in (12). First of all we will find the solution of concentration and heat equation because the solution of the velocity equation is dependent upon the solution of these two classes.

### Solution of concentration equation

The concentration equation in dimensionless form is given as;

$$\frac{\partial C(\varphi ,t)}{{\partial t}} = \frac{1}{Sc}\frac{{\partial^{2} C(\varphi ,t)}}{{\partial \varphi^{2} }} - \eta_{2} C(\varphi ,t),$$

$$C(\varphi ,0) = 0,\;\;C(0,t) = 1,\;\;{\text{and}}\;\;C(\infty ,t) = 0,$$

When we introduce the fractional operator of Yang–Abdel–Cattani the equation become as;

16 $$^{YAC} D_{t}^{\alpha } C(\varphi ,t) = \frac{1}{Sc}\frac{{\partial^{2} C(\varphi ,t)}}{{\partial \varphi^{2} }} - \eta_{2} C(\varphi ,t),$$

After applying the Laplace transform to the (16) and using (12) we get;

$$\frac{1}{{s^{\alpha + 1} }}\frac{{sL\{ C(\varphi ,t)\} - C(\varphi ,0)}}{{1 + \wp s^{ - (\alpha + 1)} }} = \frac{1}{Sc}\frac{{\partial^{2} \overline{C}(\varphi ,s)}}{{\partial \varphi^{2} }} - \eta_{2} \overline{C}(\varphi ,s),$$

After simplification it becomes;

17 $$\left( {\frac{s}{{s^{\alpha + 1} + \wp }} + \eta_{2} } \right)\overline{C}(\varphi ,s) = \frac{1}{Sc}\frac{{\partial^{2} \overline{C}(\varphi ,s)}}{{\partial \varphi^{2} }}{,}$$

with transformed IC’s and BC’s;

$$\overline{C}(\varphi ,0) = 0,\, \, \overline{C}(0,s) = \frac{1}{s},\,{\text{ and }}\overline{C}(\infty ,s) = 0,\,$$

The solution of (17) using the appropriate initial boundary conditions is given as;

18 $$\overline{C}(\varphi ,s) = \frac{1}{s}\exp \left( { - \varphi \sqrt {\frac{s.Sc}{{s^{\alpha + 1} + \wp }} + \eta_{2} .Sc} } \right){.}$$

By using Taylor’s series expansion we can write (18) in series equivalent form so that to derive the inverse Laplace transform of the function more easily. In series equivalent form it can be written as;

$$\overline{C}(\varphi ,s) = \frac{1}{s}\sum\limits_{n = 0}^{\infty } {\frac{{\left( { - \varphi \sqrt {Sc} } \right)^{n} }}{n!}\frac{{\left( {(s^{\alpha + 1} + \wp )\eta_{2} + s} \right)^{\frac{n}{2}} }}{{(s^{\alpha + 1} + \wp )^{\frac{n}{2}} }}} .$$

The required solution of the concentration field after applying inverse Laplace transform is given as;

$$C(\varphi ,t) = \sum\limits_{n = 0}^{\infty } {\frac{{\left( { - \varphi \sqrt {Sc} } \right)^{n} }}{n!}t^{\alpha n} \left( {\frac{{\left( {1 + \frac{n}{2}\eta_{2} E_{{\alpha + 1,\frac{\alpha n}{2} + 1}}^{\frac{n}{2}} (\wp t^{\alpha + 1} )} \right)}}{{\left( {E_{{\alpha + 1,\frac{\alpha n}{2} + 1}}^{\frac{n}{2}} ( - \wp t^{\alpha + 1} )} \right)}}} \right)} .$$

### Solution of temperature equation

The temperature equation in dimensionless form is as;

$$\frac{\partial T(\varphi ,t)}{{\partial t}} = - \frac{1}{\Pr }\frac{{\partial^{2} T(\varphi ,t)}}{{\partial \varphi^{2} }} - \eta_{1} T(\varphi ,t),$$

$$T(\varphi ,0) = 0,\,{\text{ T}}(0,t) = 1,\,{\text{ and T}}(\infty ,t) = 0,\,$$

When we introduce the fractional operator of Yang–Abdel–Cattani the equation become as;

19 $$^{YAC} D_{t}^{\alpha } T(\varphi ,t) = - \frac{1}{\Pr }\frac{{\partial^{2} T(\varphi ,t)}}{{\partial \varphi^{2} }} - \eta_{1} T(\varphi ,t),$$

After using the Laplace transform to the (19) and using (12) we get;

$$\frac{1}{{s^{\alpha + 1} }}\frac{{sL\{ T(\varphi ,t)\} - T(\varphi ,0)}}{{1 + \wp s^{ - (\alpha + 1)} }} = - \frac{1}{\Pr }\frac{{\partial^{2} \overline{T}(\varphi ,s)}}{{\partial \varphi^{2} }} - \eta_{1} \overline{T}(\varphi ,s),$$

After simplification it becomes;

20 $$\left( {\frac{s}{{s^{\alpha + 1} + \wp }} + \eta_{1} } \right)\overline{T}(\varphi ,s) = \frac{1}{\Pr }\frac{{\partial^{2} \overline{T}(\varphi ,s)}}{{\partial \varphi^{2} }}{,}$$

with transformed initial boundary conditions

$$\overline{T}(\varphi ,0) = 0,\;\;\overline{T}(0,s) = \frac{1}{s},\;\;{\text{and}}\;\;\overline{T}(\infty ,s) = 0,$$

The solution of (20) using the appropriate initial boundary conditions is given as;

21 $$\overline{T}(\varphi ,s) = \frac{1}{s}\exp \left( { - \varphi \sqrt {\frac{\Pr .s}{{s^{\alpha + 1} + \wp }} + \eta_{1} .\Pr } } \right){,}$$

By using Taylor’s series expansion we can write (21) in series equivalent form so that to derive the inverse Laplace transform of the function more easily. In series equivalent form it can be written as;

$$\overline{T}(\varphi ,s) = \frac{1}{s}\sum\limits_{n = 0}^{\infty } {\frac{{\left( { - \varphi \sqrt {\Pr } } \right)^{n} }}{n!}\frac{{\left( {(s^{\alpha + 1} + \wp )\eta_{1} + s} \right)^{\frac{n}{2}} }}{{(s^{\alpha + 1} + \wp )^{\frac{n}{2}} }}} ,$$

The required solution of the temperature field after applying inverse Laplace transform is given as;

$$T(\varphi ,t) = \sum\limits_{n = 0}^{\infty } {\frac{{\left( { - \varphi \sqrt {\Pr } } \right)^{n} }}{n!}t^{\alpha n} \left( {\frac{{1 + \frac{n}{2}\eta_{1} E_{{\alpha + 1,\frac{\alpha n}{2} + 1}}^{\frac{n}{2}} (\wp t^{\alpha + 1} )}}{{E_{{\alpha + 1,\frac{\alpha n}{2} + 1}}^{\frac{n}{2}} ( - \wp t^{\alpha + 1} )}}} \right)} .$$

### Solution of velocity equation

The velocity equation in dimensionless form is given as;

$$\frac{\partial v(\varphi ,t)}{{\partial t}} = \frac{1}{\lambda }\frac{{\partial^{2} v(\varphi ,t)}}{{\partial \varphi^{2} }} - Mv(\varphi ,t) + GrT(\varphi ,t) + GmC(\varphi ,t),$$

$$v(\varphi ,0) = 0,\;\;{\text{v}}(0,t) = 1,\;\;{\text{and}}\;\;{\text{v}}(\infty ,t) = 0,$$

When we introduce the fractional operator of Yang–Abdel–Cattani the equation become as;

22 $$^{YAC} D_{t}^{\alpha } v(\varphi ,t) = \frac{1}{\lambda }\frac{{\partial^{2} v(\varphi ,t)}}{{\partial \varphi^{2} }} - Mv(\varphi ,t) + GrT(\varphi ,t) + GmC(\varphi ,t),$$

After using the Laplace transform to the (20) and using (12) we get;

$$\frac{1}{{s^{\alpha + 1} }}\frac{{sL\{ v(\varphi ,t)\} - v(\varphi ,0)}}{{1 + \wp s^{ - (\alpha + 1)} }} = \frac{1}{\lambda }\frac{{\partial^{2} \overline{v}(\varphi ,t)}}{{\partial \varphi^{2} }} - M\overline{v}(\varphi ,t) + Gr\overline{T}(\varphi ,t) + Gm\overline{C}(\varphi ,t),$$

After simplification it becomes;

23 $$\frac{s}{{s^{\alpha + 1} + \wp }}\overline{v}(\varphi ,s) = \frac{1}{\lambda }\frac{{\partial^{2} \overline{v}(\varphi ,t)}}{{\partial \varphi^{2} }} - M\overline{v}(\varphi ,t) + Gr\overline{T}(\varphi ,t) + Gm\overline{C}(\varphi ,t),$$

with transformed initial boundary conditions

$$\overline{v}(\varphi ,0) = 0,\;\;{\overline{\text{v}}}(0,s) = \frac{1}{s},\;\;{\text{and}}\;\;{\overline{\text{v}}}(\infty ,s) = 0,$$

The solution of (23) using the appropriate initial boundary conditions is given as;

24 $$\begin{aligned} & \overline{v}(\varphi ,s) = \overline{v}_{c} (\varphi ,s) + \overline{v}_{p} (\varphi ,s), \\ & \overline{v}_{c} (\varphi ,s) = A\exp \left( { - \varphi \sqrt {\frac{\lambda .s}{{s^{\alpha + 1} + \wp }} + M.\lambda } } \right){,} \\ \end{aligned}$$

and

$$\begin{aligned} \overline{v}_{p} (\varphi ,s) & = \left( {\left( {\frac{{s^{\alpha + 1} + \wp }}{s}} \right)\left( {\frac{Gr}{{\left( {\Pr - \lambda } \right)s + \left( {\eta_{1} .\Pr - M \cdot \lambda } \right)\left( {s^{\alpha + 1} + \wp } \right)}}} \right)} \right)\exp \left( { - \varphi \sqrt {\frac{\Pr .s}{{s^{\alpha + 1} + \wp }} + \eta_{1} .\Pr } } \right) \\ & \quad - \left( {\left( {\frac{{s^{\alpha + 1} + \wp }}{s}} \right)\left( {\frac{Gm}{{\left( {Sc - \lambda } \right)s + \left( {\eta_{2} .Sc - M \cdot \lambda } \right)\left( {s^{\alpha + 1} + \wp } \right)}}} \right)} \right)\exp \left( { - \varphi \sqrt {\frac{s.Sc}{{s^{\alpha + 1} + \wp }} + \eta_{2} .Sc} } \right){.} \\ \end{aligned}$$

the value of constant A is given by;

$$A = \frac{1}{s} - \left( {\left( {\frac{Gr}{{\left( {\Pr - \lambda } \right)s + \left( {\eta_{1} .\Pr - M \cdot \lambda } \right)\left( {s^{\alpha + 1} + \wp } \right)}}} \right) - \left( {\frac{Gm}{{\left( {Sc - \lambda } \right)s + \left( {\eta_{2} .Sc - M \cdot \lambda } \right)\left( {s^{\alpha + 1} + \wp } \right)}}} \right)} \right){,}$$

The general solution for the velocity equation is given as;

25 $$\begin{aligned} \overline{v}(\varphi ,s) & = \left[ {\frac{1}{s} - \left( \begin{gathered} \left( {\frac{{s^{\alpha + 1} + \wp }}{s}} \right)\left( {\frac{Gr}{{\left( {\Pr - \lambda } \right)s + \left( {\eta_{1} .\Pr - M \cdot \lambda } \right)\left( {s^{\alpha + 1} + \wp } \right)}}} \right) \hfill \\ - \left( {\frac{{s^{\alpha + 1} + \wp }}{s}} \right)\left( {\frac{Gm}{{\left( {Sc - \lambda } \right)s + \left( {\eta_{2} .Sc - M \cdot \lambda } \right)\left( {s^{\alpha + 1} + \wp } \right)}}} \right) \hfill \\ \end{gathered} \right)} \right]\exp \left( { - \varphi \sqrt {\frac{\lambda .s}{{s^{\alpha + 1} + \wp }} + M.\lambda } } \right) \, \\ & \quad { + }\left( {\frac{{s^{\alpha + 1} + \wp }}{s}} \right)\left( {\frac{Gr}{{\left( {\Pr - \lambda } \right)s + \left( {\eta_{1} .\Pr - M \cdot \lambda } \right)\left( {s^{\alpha + 1} + \wp } \right)}}} \right)\exp \left( { - \varphi \sqrt {\frac{\Pr .s}{{s^{\alpha + 1} + \wp }} + \eta_{1} .\Pr } } \right) \, \\ & \quad - \left( {\frac{{s^{\alpha + 1} + \wp }}{s}} \right)\left( {\frac{Gm}{{\left( {Sc - \lambda } \right)s + \left( {\eta_{2} .Sc - M \cdot \lambda } \right)\left( {s^{\alpha + 1} + \wp } \right)}}} \right)\exp \left( { - \varphi \sqrt {\frac{s.Sc}{{s^{\alpha + 1} + \wp }} + \eta_{2} .Sc} } \right){.} \\ \end{aligned}$$

Equation (25) represents the general solution of the velocity equation.

Where

The inverse Laplace transform of the function is given as;

$$\begin{aligned} v_{1} (\varphi ,t) = L^{ - 1} \left\{ {\overline{v}_{1} (\varphi ,s)} \right\} & = L^{ - 1} \left\{ {\exp \left( { - \varphi \sqrt {\frac{\lambda .M}{{s^{\alpha + 1} + \wp }} + \lambda .M} } \right)} \right\} \\ & = L^{ - 1} \left\{ {\sum\limits_{n = 0}^{\infty } {\frac{{\left( { - \varphi \sqrt \lambda } \right)^{n} }}{n!}\frac{{\left( {(s^{\alpha + 1} + \wp )M + s} \right)^{\frac{n}{2}} }}{{(s^{\alpha + 1} + \wp )^{\frac{n}{2}} }}} } \right\} \\ & = \sum\limits_{n = 0}^{\infty } {\frac{{\left( { - \varphi \sqrt \lambda } \right)^{n} }}{n!}t^{\alpha n} \left( {\frac{{1 + \frac{n}{2}ME_{{\alpha + 1,\frac{\alpha n}{2} + 1}}^{\frac{n}{2}} (\wp t^{\alpha + 1} )}}{{E_{{\alpha + 1,\frac{\alpha n}{2} + 1}}^{\frac{n}{2}} ( - \wp t^{\alpha + 1} )}}} \right)} \\ \end{aligned}$$

$$\begin{aligned} v_{2} (\varphi ,t) = L^{ - 1} \left\{ {\overline{v}_{2} (\varphi ,s)} \right\} & = L^{ - 1} \left\{ {\exp \left( { - \varphi \sqrt {\frac{\Pr .s}{{s^{\alpha + 1} + \wp }} + \eta_{1} .\Pr } } \right)} \right\} \\ & = L^{ - 1} \left\{ {\sum\limits_{n = 0}^{\infty } {\frac{{\left( { - \varphi \sqrt {\Pr } } \right)^{n} }}{n!}\frac{{\left( {(s^{\alpha + 1} + \wp )\eta_{1} + s} \right)^{\frac{n}{2}} }}{{(s^{\alpha + 1} + \wp )^{\frac{n}{2}} }}} } \right\} \\ & = \sum\limits_{n = 0}^{\infty } {\frac{{\left( { - \varphi \sqrt {\Pr } } \right)^{n} }}{n!}t^{\alpha n} \left( {\frac{{1 + \frac{n}{2}\eta_{1} E_{{\alpha + 1,\frac{\alpha n}{2} + 1}}^{\frac{n}{2}} (\wp t^{\alpha + 1} )}}{{E_{{\alpha + 1,\frac{\alpha n}{2} + 1}}^{\frac{n}{2}} ( - \wp t^{\alpha + 1} )}}} \right)} \\ \end{aligned}$$

$$\begin{aligned} v_{3} (\varphi ,t) = L^{ - 1} \left\{ {\overline{v}_{3} (\varphi ,s)} \right\} & = L^{ - 1} \left\{ {\exp \left( { - \varphi \sqrt {\frac{Sc.s}{{s^{\alpha + 1} + \wp }} + \eta_{2} .Sc} } \right)} \right\} \\ & = L^{ - 1} \left\{ {\sum\limits_{n = 0}^{\infty } {\frac{{\left( { - \varphi \sqrt {Sc} } \right)^{n} }}{n!}\frac{{\left( {(s^{\alpha + 1} + \wp )\eta_{2} + s} \right)^{\frac{n}{2}} }}{{(s^{\alpha + 1} + \wp )^{\frac{n}{2}} }}} } \right\} \\ & = \sum\limits_{n = 0}^{\infty } {\frac{{\left( { - \varphi \sqrt {Sc} } \right)^{n} }}{n!}t^{\alpha n} \left( {\frac{{1 + \frac{n}{2}\eta_{2} E_{{\alpha + 1,\frac{\alpha n}{2} + 1}}^{\frac{n}{2}} (\wp t^{\alpha + 1} )}}{{E_{{\alpha + 1,\frac{\alpha n}{2} + 1}}^{\frac{n}{2}} ( - \wp t^{\alpha + 1} )}}} \right)} \\ \end{aligned}$$

$$\begin{aligned} v_{4} (\varphi ,t) & = L^{ - 1} \left\{ {\frac{{s^{\alpha + 1} + \wp }}{{s^{2} }}} \right\} \\ & { = }\frac{1}{{t^{\alpha } \Gamma (1 - \alpha )}} + \wp t \\ \end{aligned}$$

The equation given in (26) represents the exact solution of the velocity equation after applying the inverse Laplace transform to the analytical solution of the velocity equation given in (25) by using the above inverse transforms of the exponential functions;

26 $$\begin{aligned} v(\varphi ,t) & = \left[ {1 - \left( \begin{gathered} v_{4} (\varphi ,t)*\left( {\frac{Gr}{{\left( {\Pr - \lambda } \right) + \left( {\eta_{1} .\Pr - M \cdot \lambda } \right)}}} \right) \hfill \\ - v_{4} (\varphi ,t)*\left( {\frac{Gm}{{\left( {Sc - \lambda } \right)s + \left( {\eta_{2} .Sc - M \cdot \lambda } \right)\left( {s^{\alpha + 1} + \wp } \right)}}} \right) \hfill \\ \end{gathered} \right)} \right]{*}v_{1} (\varphi ,t) \, \\ & \quad { + }\left( {v_{4} (\varphi ,t)*\left( {\frac{Gr}{{\left( {\Pr - \lambda } \right) + \left( {\eta_{1} .\Pr - M \cdot \lambda } \right)}}} \right)*v_{2} (\varphi ,t)} \right) \\ & \quad - \left( {v_{4} (\varphi ,t)\left( {\frac{Gm}{{\left( {Sc - \lambda } \right) + \left( {\eta_{2} .Sc - M \cdot \lambda } \right)}}} \right)*v_{3} (\varphi ,t)} \right){.} \\ \end{aligned}$$

## Results and discussion

In this research article we analyzed the exact analytical solution of the problem of the fractional Casson fluid by using the new fractional operator with exponential kernel of Rabotnov i.e. Yang–Abdel–Cattani operator of fractional derivative. The influence of heat source, magnetic hydrodynamics and chemical reactions on the flow of fractional Casson fluid through a flat plate is studied in this article. For the sake of a better interpretation of the rheological behavior of Casson fluid we have used the new operator of fractional order with exponential kernel of Rabotnov known as Yang-Abdel Cattani operator of fractional order. The Yang-Abdel Cattani operator of fractional order can describe the memory effects more suitably than the other fractional operators. By making use of the technique of Laplace integral transformation we have find the exact analytical solution of the problem in the Mittag–Leffler forms, for all the three governing equations i.e. Velocity, energy and concentration equation.

**Figure 2:**
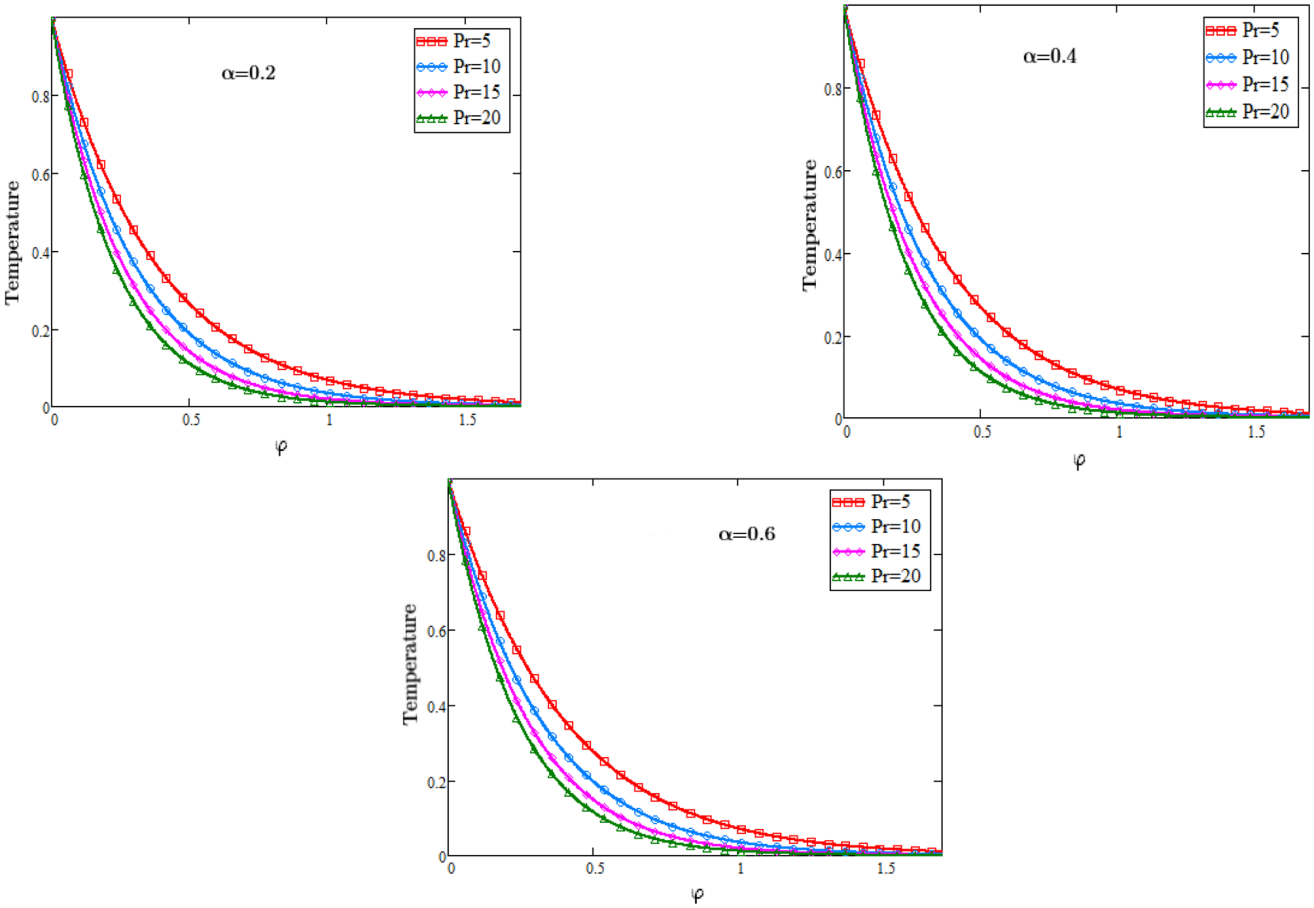
Temperature Profiles of dimensionless Prandtl number.

To analyze the physical importance of different fluid parameters such as Sc, Pr, MHD and alpha on the temperature, concentration and velocity class are presented through graphs. Figure 2 is sketched to check the effects of eta ${\eta }_{1}$ for temperature profile against φ, from which it is observed that by increasing the value of eta ${\eta }_{1}$ temperature decreases, because the consistency of thermal boundary layer decreases with the increasing values of parameter eta ${\eta }_{1}$. The role of heat source in a fluid transport is to increase its thermal conductivity and when the heat sources and all the heat fluxes are constant then in that case we consider the free convection heat transfer.

**Figure 3:**
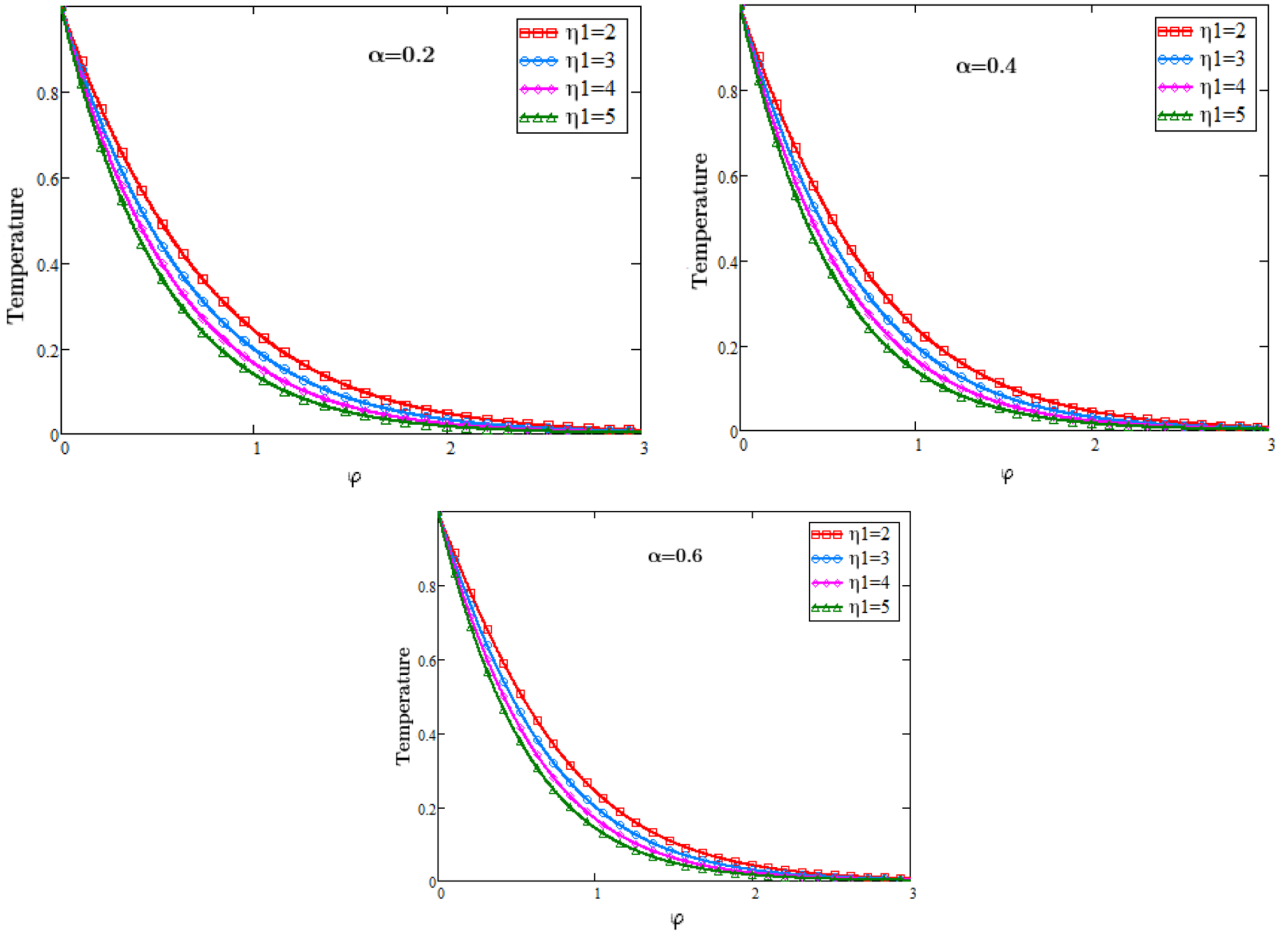
Temperature Profiles of dimensionless parameter of heat source.

**Figure 4:**
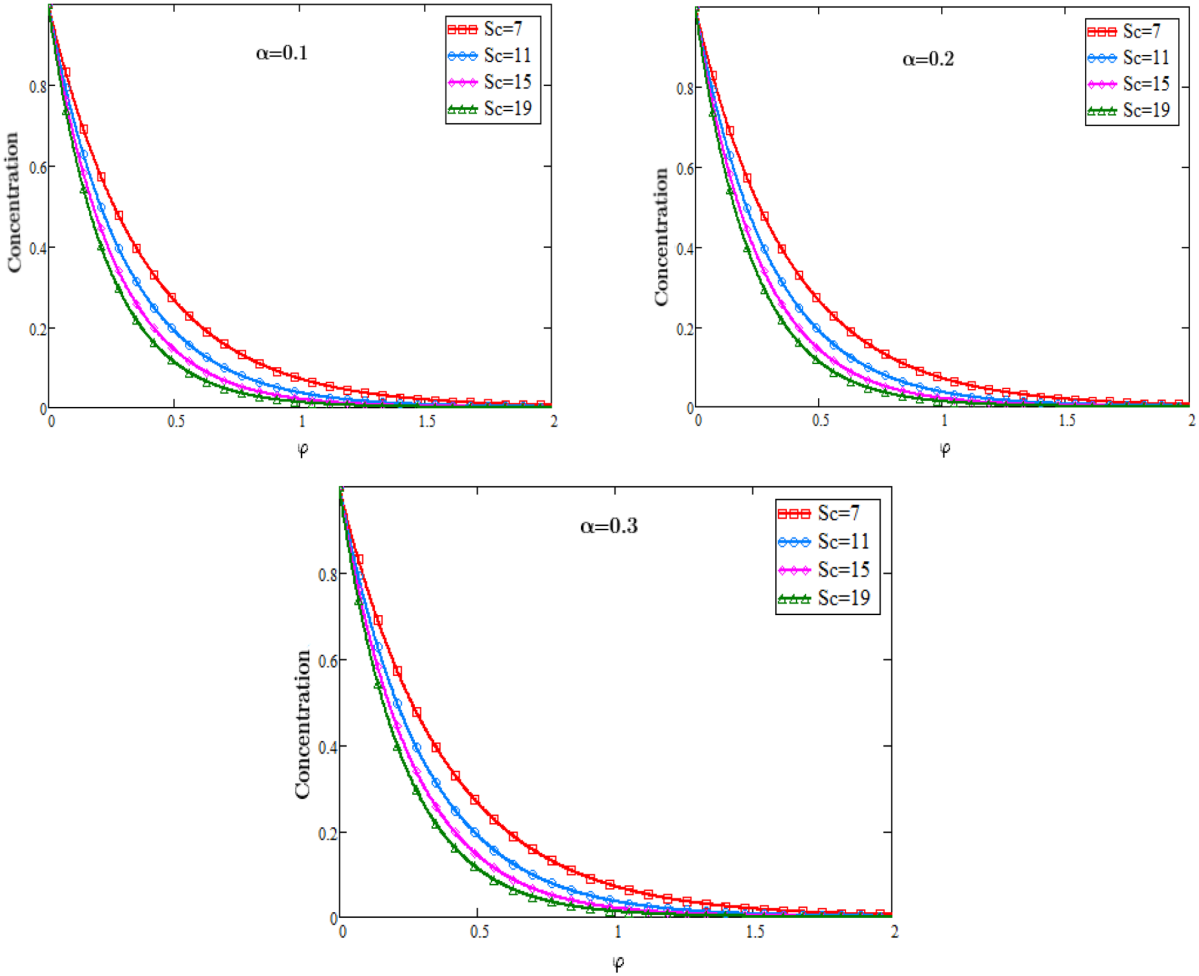
Concentration profiles for dimensionless Schmidt number.

The graphs in Fig. 3 describe the impacts of Prandtl number on the temperature of the fluid against φ and an important impact on the temperature of the fluid in the boundary layer is analyzed. As we know that when the Prandtl number increases the heat conduction decreases since it is the ratio of Kinematic viscosity to heat conductivity. Due to decrease in the heat conductivity with the rising values of Prandtl number, consequently temperature of the fluid decreases. In physical sense the Prandtl number describes the relative thickness of the momentum and thermal boundary layer in heat transfer problem. The graphs in Fig. 4 represents the impacts of Schmidt number on the concentration of the fluid against φ and an important effect on the diffusion rate of the mass of the fluid in the boundary layer is analyzed. As we know that when the Schmidth number increases the diffusion rate decreases since it is the ratio of Kinematic viscosity to the mass diffusion rate. Due to decrease in the diffusion rate with the rising values of the Schmidth number, consequently the concentration of the fluid decreases. In physical sense the Schmidth number characterizes the fluid flow with simultaneous momentum and mass diffusion convection problems.

**Figure 5:**
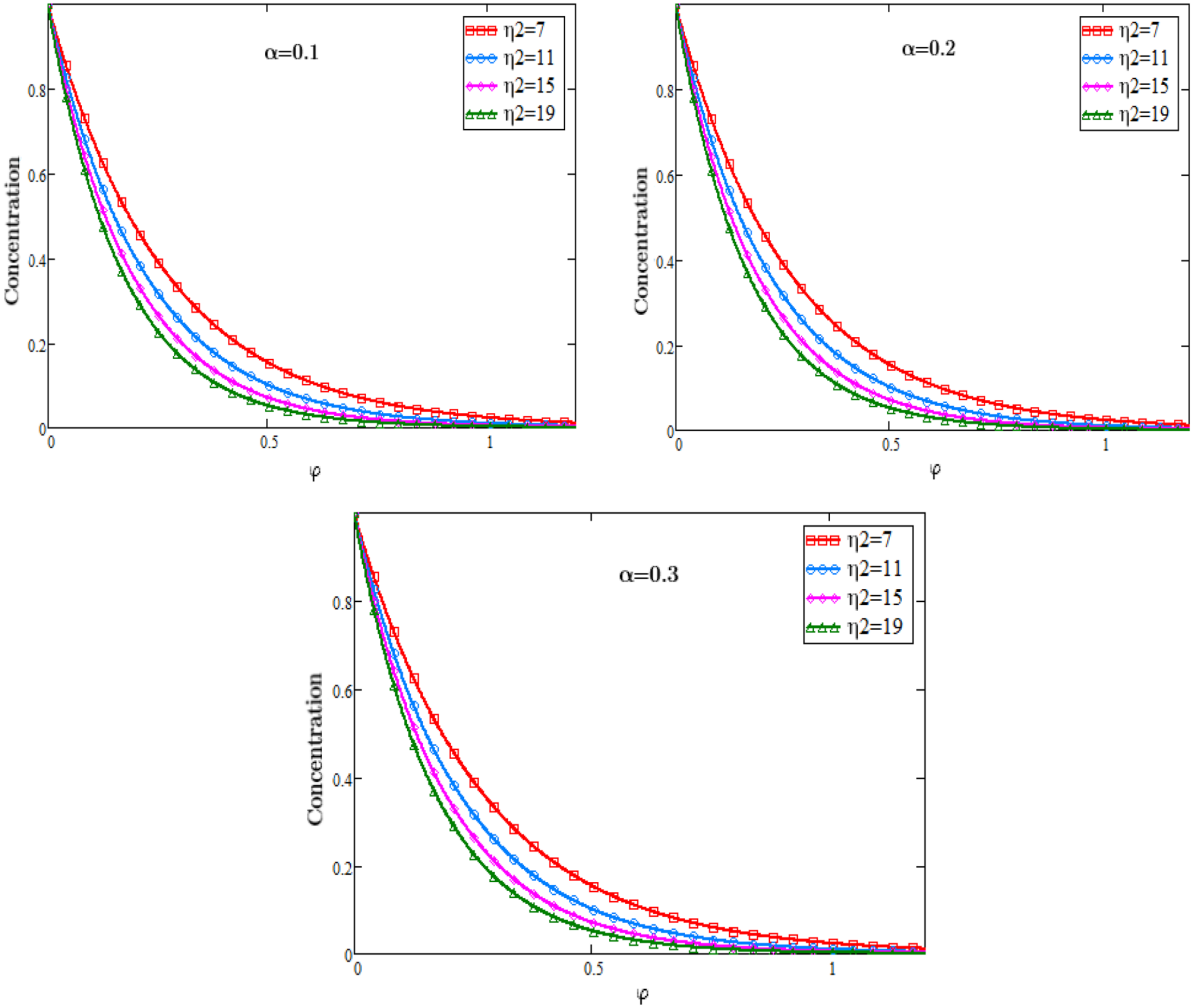
Profiles of concentration for dimensionless parameter of chemical reaction.

The graphs in Fig. 5 represents the impacts of dimensionless parametre of chemical reaction ${\eta }_{2}$ on the concentration of the fluid against φ and an important effect on the diffusion rate of mass of the fluid in the boundary layer is analyzed. With the rising values of the parameter ${\eta }_{2}$ the concentration of the fluid decreases. As the values of the parameter of the chemical reaction ${\eta }_{2}$ increases, it consequently increases the rate of the reaction due to which the concentration of the reactant decreases which causes a decrease in the rate of the diffusion.

**Figure 6:**
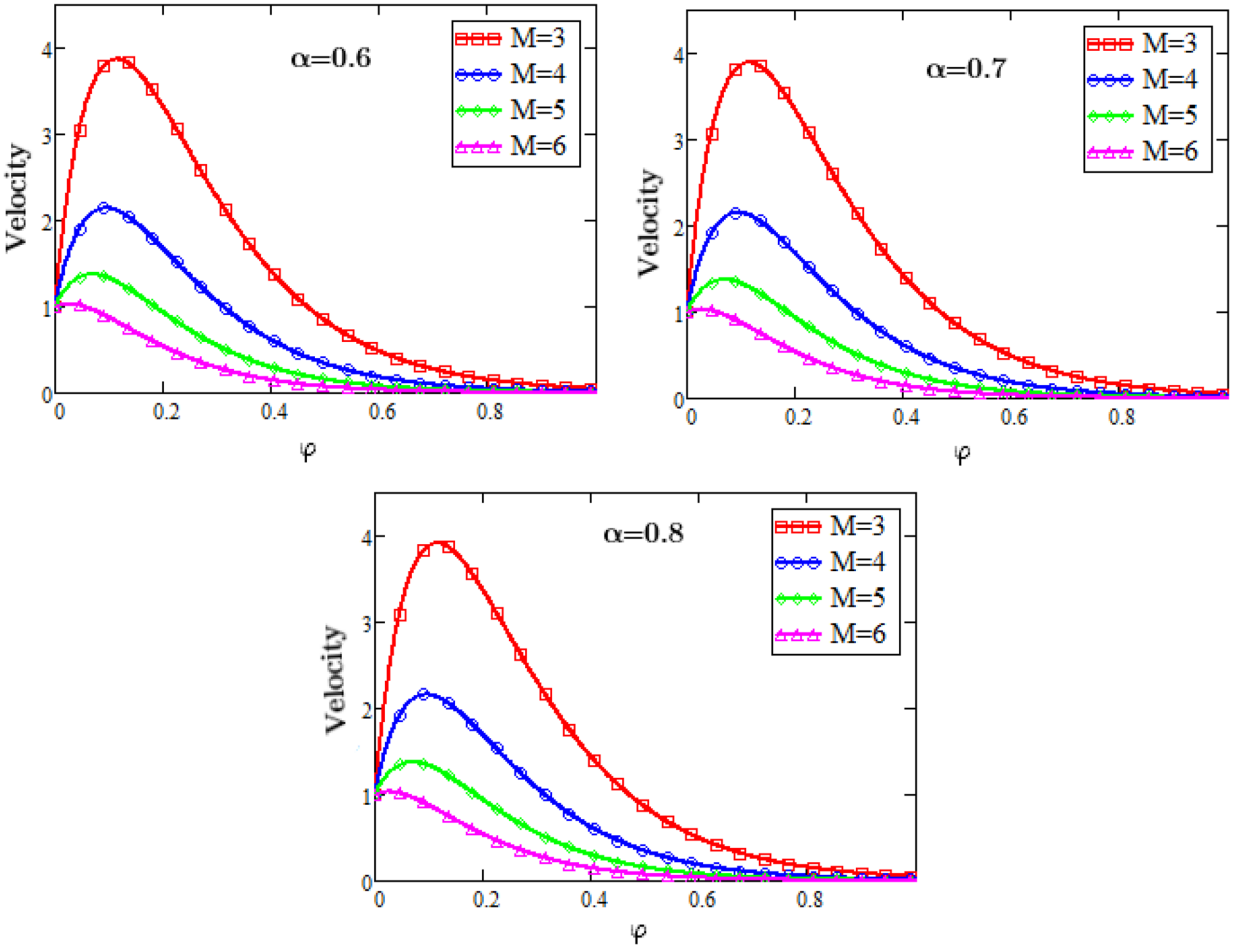
Velocity Profiles for dimensionless parameter of MHD.

**Figure 7:**
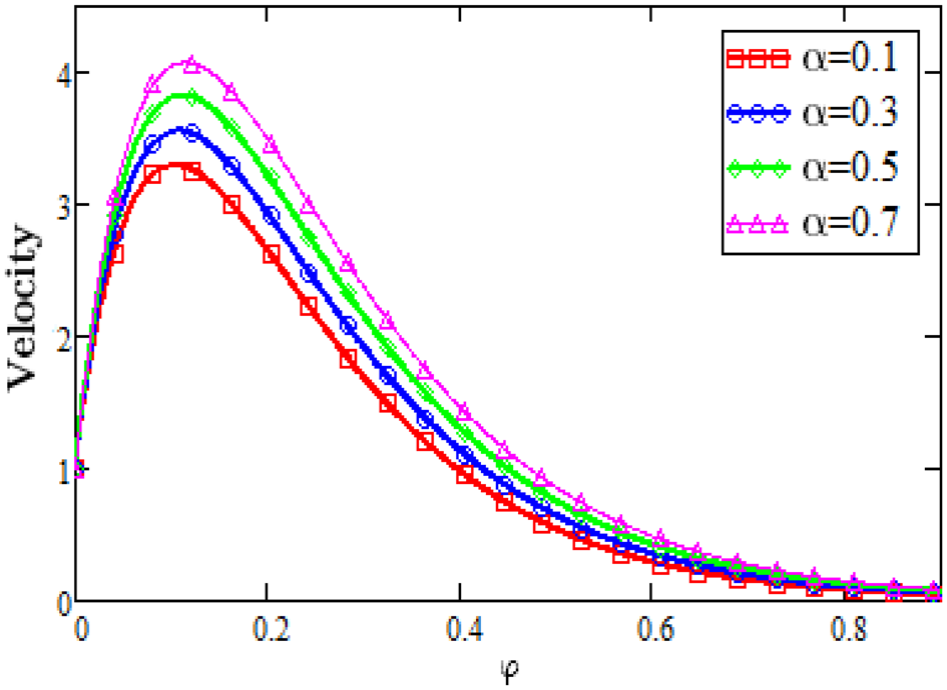
Velocity profiles for dimensionless fractional parameter alpha.

Figure 6 represents the graphs of velocity profiles for the effects of the magneto hydrodynamics against φ. With the increase in the values of the magnetic hydrodynamics results in the increase of the resistive force that is known as Lorentz force which increases the drag during the flow due to this increasing drag force the velocity of the fluid decreases. So with the accelerating values of the parameter of magneto hydrodynamics M the velocity of the fluid decreases. Figure 7 shows that, as the values of the fractional parameter alpha rises the velocity of the fluid rises, because the increase in the fractional parameter alpha causes decrease in the boundary layer thickness.

### Temperature profiles

Figure 4.

### Concentration profiles

Figure 5.

### Velocity profiles

Figure 6.

## Conclusion

This research paper is dedicated to investigates exact analytical solution for problem of fractional Casson fluid by using the new fractional operator with exponential kernel of Rabotnov i.e. Yang–Abdel–Cattani operator. The effects of magnetic hydrodynamics, chemical reactions and heat source on the flow of fractional Casson fluid through a verticle plate are studied in this article. By using the Buckingham Pi theorem we obtained the dimensionless form of problem. For the sake of a better explanation of the rheological behavior of Casson fluid we have used the new fractional operator with Rabotnov exponential kernel known as Yang-Abdel Cattani operator. The Yang-Abdel Cattani operator of fractional order can describe the memory effects more suitably than the other fractional operators. The Laplace transform is used to find the exact analytical solution of the problem in the terms of Mittag–Leffler functions, for all the three governing equations i.e. Velocity, energy and concentration equation. The results achieved in the results and discussion section can be concluded as;

- The Prandtl number describes controls the relative thickness of momentum and temperature boundary layer. The temperature of the fluid decreases with the increasing values of the Prandtl number.
- In physical sense the Schmidth number characterizes the fluid flow with simultaneous momentum and mass diffusion convection problems. The rate of diffusion decreases with the increasing values of the Schmidt number.
- The rate of mass diffusion decreases with the increasing values of the parameter of chemical reaction i.e. eta.
- The fluid’s velocity decreases as effects of MHD increasing.
- The fluid’s velocity increases as the values of alpha increases.

### Future recommendations

- The current work can be extended to study the combine effects of heat source and radiation.
- This can also be extended for the combine effects of porosity and MHD.
- The same problem can be solved for considering slip wall condition at velocity boundary layer.
- The same problem can be solved for considering Newtonian heating source at Temperature boundary layer.
- The same problem can be solved for considering exponentially varying concentration at Concentration boundary layer.

## Data Availability

The datasets used and analyzed during the current study available from the corresponding author on reasonable request.

## List of symbols

*v*: Velocity (L T^−1^)
*v*: Viscosity (kinematic) (L^2^ T^−1^)
$\beta_{T}$: Thermal expansion coefficient in volume (θ^−1^)
$T_{\infty }$: The fluid temperature away from plate
*k*: Conductivity of heat (M L T^−3^ θ^−1^)
*G*_*m*_: Mass Grashof
*C*_*w*_: At plate fluid concentration
$C_{\infty }$: Far away fluid concentration from plate
$\rho$: Density of the fluid (M L^−3^)
*g*: Acceleration (gravity)
*λ*: Porosity parameter
*T*: Temperature (θ)
*μ*: $$\mathrm{Viscosity }(\mathrm{dynamic})$$ (M L^−1^ T^−1^)
Pr: Prandtl number $\left( {\frac{{\mu C_{p} }}{k}} \right)$
*C*_*p*_: Constant-pressure specific heat (L^2^ M T^−1^ θ^−1^)
*t*: Time (T)
*α*: Fractional parameter
*T*_*w*_: At the plate fluid temperature
*C*: Fluid concentration
*Gr*: Thermal Grashof
η: Chemical reaction parameter
$\gamma$: Grade-second parameter (dimensionless)
*s*: Laplace transform parameter
